# Fear of COVID-19, risk perception and preventive behavior in health workers: a cross-sectional analysis in middle-income Latin American countries

**DOI:** 10.3389/fpubh.2023.1171246

**Published:** 2023-06-15

**Authors:** César Antonio Bonilla-Asalde, Oriana Rivera-Lozada, Miguel Ipanaqué-Zapata, Elvis Siprian Castro-Alzate, Robinson Pacheco-Lopez, Isabel Cristina Rivera-lozada, Félix Chong, Lucrecia Ramírez Sagastume

**Affiliations:** ^1^Professional School of Human Medicine, Universidad Privada San Juan Bautista, Lima, Peru; ^2^South American Center for Education and Research in Public Health, Universidad Privada Norbert Wiener, Lima, Peru; ^3^Instituto de Investigación, Capacitación y Desarrollo Psicosocialy Educativo: PSYCOPERU, Lima, Peru; ^4^Academic Program of Occupational Therapy, Academic Program of Doctorate in Health, Universidad del Valle, Cali, Cauca, Colombia; ^5^Universidad Libre, Cali, Cauca, Colombia; ^6^Department Economy, Universidad del Cauca, Popayán, Cauca, Colombia; ^7^Ministry of Health of Ecuador, Quito, Pichincha, Ecuador; ^8^Ministry of Health of Guatemala, Lima, Guatemala

**Keywords:** COVID-19, SARS-CoV-2, health personnel, fear to COVID-19, behavior

## Abstract

The aim of this study was to examine the association between fear of COVID-19 and risk perception with preventive behavior in health professionals from four Latin American countries. An analytical cross-sectional study was conducted. Health professionals with on-site care in Colombia, Ecuador, Guatemala, and Peru were surveyed. Information was collected through an online self-report questionnaire. The main variables were preventive behavior as the dependent variable and fear of COVID-19 and risk perception as independent variables. Linear regression was used, and unstandardized beta coefficient and value of ps were calculated. Four hundred and thirty-five health professionals were included, the majority were aged 42 years or older (45.29, 95%CI: 40.65–50.01) and female (67.82, 95%CI: 63.27–72.05). It was shown that the greater the fear of COVID-19, the greater the preventive behavior of COVID-19 infection (*B* = 2.21, *p* = 0.002 for total behavior; *B* = 1.12, *p* = 0.037 for additional protection at work; *B* = 1.11, *p* < 0.010 for hand washing). The risk perception of COVID-19 infection had a slight direct relationship with preventive behaviours (*B* = 0.28, *p* = 0.021 for total behavior; *B* = 0.13, *p* = 0.015 for hand washing), with the exception of the preventive behavior of using additional protection at work (*p* = 0.339). We found that fear and risk perception are associated with increased practice of hand washing and use of additional protection at work. Further studies are required on the influence of working conditions, job performance and the occurrence of mental health problems in frontline personnel with regard to COVID-19.

## Introduction

Since the beginning of the pandemic, COVID-19 has caused significant damage to health systems around the world, including financial, material and, mainly, human lives losses (1, 2). All this, despite the strict measures promoted by the authorities to prevent transmission (3), such as strict social distancing, lockdowns and educational campaigns (4). In addition, the accelerated speed with which the virus spread created challenges in health care systems that forced health care workers to deal with both clinical and non-clinical stressors (5). This scenery is even more challenging in Latin America, where there are other points to concern, such as deep social inequalities, economic instability, and deficient health care services (6).

Fear is one of the first mental reactions appearing in an epidemic. This phenomenon allows us to survive and confront the unknown (7). This situation impacts HCWs (8–10), adding enormous psychological pressure. Although, it may be beneficial because it encourages them to follow preventive measures such as hand washing and social distancing (11). However, the exposition to fear for long periods can involve pathological mechanisms, affecting the well-being and the ability to provide adequate treatment and care (12). The context of COVID-19 was complex and triggered fear in the global population, especially WHCs. A systematic review concluded that WHCs have 19.51 as pooled mean score according to the FCV-19S scale. This value was the highest score in comparison with the general population and university students (13). The increasing mortality and morbidity associated with COVID-19 (14) have caused fear of acquiring the disease and, above all, of dying for it (15–17). Additionally to this, HCWs confront the fear of bringing the virus to family members (8, 18) the constant loss of colleagues to the disease (19), and the shortage of personal protective equipment (PPE).

Evidence from previous outbreaks (20–22), together with evidence in the COVID-19 pandemic (23–25), suggests that these triggers have significant short- and long-term effects on the mental health of healthcare workers. Furthermore, fear of COVID-19 correlates with other mental illnesses such as anxiety, traumatic stress, distress (strong association), and depression (moderate association) (26). Some studies have shown a potential association between fear of COVID-19 and suicidal thoughts and insomnia. (27).

Therefore, the impact of the COVID-19 pandemic on the mental health of HCWs is multiple and has potential long-term effects that the healthcare system may face going forward. This is why it is very important to take care of the mental health of these professionals (14). However, there is still little evidence of the relationship between these outcomes on a region such a Latin America. Therefore, the aim of this study was to examine the association between fear of COVID-19 and risk perception with preventive behavior in health professionals from four Latin American countries.

## Materials and methods

### Study design and area

An observational analytical cross-sectional study was carried out during the COVID-19 pandemic in health personnel from 4 Latin American (LA) countries: Colombia, Ecuador, Guatemala, and Peru. Latin America is made up of 20 countries, with notable cultural, economic and political differences (28). For example, according to gross national income (GNI), Colombia, Ecuador, Guatemala and Peru are upper middle-income countries (29).

### Sample size

A total of 481 health professionals with on-site care in the 4 LA countries (Colombia, Ecuador, Guatemala, and Peru) were surveyed, using snowball sampling due to the difficult access to this population in times of COVID-19. Snowball sampling is a method commonly used in research to generate a network of participants through referrals from contacts who specialize in the study’s topic. In the context of COVID-19, the accessibility to the study population was hindered by isolation measures and preventive restrictions. Therefore, snowball sampling was utilized to overcome these challenges and identify eligible participants for the study. The inclusion criteria for this study are to be a physician, nurse, or other health professional providing care in person, and to be 18 years of age or older, and to agree to participate in the study by signing the informed consent form, and to complete at least 50% of the questionnaire. From the 481 participants, 46 health professionals were excluded due to missing data, resulting in a final sample of 435 (90.443%) participants distributed in Colombia (*n* = 79), Ecuador (*n* = 121), Guatemala (*n* = 80) and Peru (*n* = 155).

### Study variables and instruments

The main study variables were preventive behavior as the dependent variable, fear of COVID-19 and risk perception as independent variables. Preventive behavior was obtained from 5 self-reported items about protective attitudes toward COVID-19 grouped according to the use of protection additional to the mask (3 items) and hand washing (2 items). The items use a Likert scale with 5 answer categories (0 = Rarely, up to 4 = Always), with final scores for the variable ranging from 0 to 20 points (additional mask use = 0 to 12 points and hand washing = 0 to 8 points).

Fear of COVID-19 was obtained from 3 self-report items about the fear of becoming infected, infecting one’s family, and dying from COVID-19, where these items had a dichotomous response scale (0 = No; 1 = Yes), with final scores ranging from 0 to 3 points.

The risk perception of COVID-19 was obtained from 4 self-report items about the existence of risk situations of direct contact with patients in care of this disease within the work environment. The items had a dichotomous response scale (0 = No; 1 = Yes), with a final score of 0 to 4 points.

All the items of the variables were housed in the supplementary section (Appendix A). Likewise, the variables used had item reliability values (α, KR-20, and Omega >0.50) and factorial structure that were adequate for the development of this study (Appendix B), as well as acceptable values of goodness of fit indicators obtained from confirmatory factor analysis (CFI > 0.90, TLI > 0.90, RMSEA<0.08, SRMR<0.08) (Appendix C).

The study covariates were age in tertiles (21 to 33, 34 to 41 and 42 or more), gender (male and female), civil status (married/cohabitant, single and others), number of children (no children, 1 child, 2 or more children), work time (in years), and mental exhaustion (No and Yes).

### Procedure

During the period between March and July 2021, health professionals with on-site care (physicians, nurses, rehabilitators, among others) were invited to participate through the Ministries of Health of the participating countries. It was important to assess the variables among healthcare professionals in the 4 countries in which data were collected, as the period from March to July 2021 reflected critical points of COVID-19 infection and mortality cases in these countries (30–33). The research team was contacted to inform about the objective of the study and to request their voluntary participation. The information was collected through the Google Forms ® platform, with an average duration of 10 min for completion. The authors ensured that the participants truly determined COVID-19 health professionals through a virtual process of presenting their work cards and the information related to their work area and the daily activities they carry out; all this information was verified before submitting the Google Form. Finally, those who completed the form were asked to refer other possible participants until the study sample was reached.

### Statistical analysis

The analysis of this study began by answering to the characterization of the main variables and covariates by reporting frequency/percentages or mean/standard deviation tables, depending on the type of variable involved. Then, in order to identify whether there were significant differences according to countries, the Chi-Square, Fisher’s Exact or ANOVA test was used, as appropriate, and for the latter, the Tukey *post hoc* test was performed to identify the country with the best scores obtained.

To answer to the aim of examining the association of fear of COVID-19 and risk perception with preventive behavior (dimensions and total) of health professionals, we used linear regression, presenting two models with unstandardized coefficient and value of ps. The first or crude model examines separately the independent variables and covariates against preventive behavior. In the final or adjusted model, a pooled model was presented with all the main independent variables and covariates that were significant in the crude model. In both models the adjustment according to country was used and the variables were significant with a *p* < 0.05. To perform a combined analysis of all countries, we first examined the measurement invariance analysis for each scale, which confirmed that the data had a similar response pattern across countries (Δ < 0.010) (Appendix D).These analyses were performed in the Stata 15.0 software (StataCorp, 2017) (34).

Additionally, for the generation of variables, reliability was taken into account through Cronbach’s alpha and internal construct validity through exploratory factor analysis using the Robust Maximum Likelihood Estimator (MLR) with rotation reporting their factor loadings (Appendix B). Cronbach’s alpha and factor loadings are adequate with values greater than 0.80 and 0.49, respectively, (35). These analyses were performed using the Rstudio software (Rstudio®, Boston, MA, United States).

### Ethical aspects

Participation was voluntary, anonymous, and written informed consent was provided within the questionnaire at the beginning of the study. The ethical guidelines of the Helsinki Declaration were followed, and the information protocol was approved by the ethics committee of the Norbert Wiener University issued in the Register Report No. 085-2020.

## Results

### Participants

The characteristics of the 435 health professionals were that the majority were 42 years of age or older (45.29, 95% CI: 40.65–50.01), female (67.82, 95% CI: 63.27–72.05), with marital status married/cohabiting (57.24, 95%CI: 52.52–61.82), with no children (43.91, 95%CI: 39.30–48.63), with a mean working time of 7.29 years (SD = 6.92) and existence of mental exhaustion (90.34, 95%CI: 87.18–92.79) (Table 1).

**Table 1:** Comparison of the characteristics of the study sample, fear of COVID-19, risk perception and preventive behaviours among health personnel according to countries.

Variables	Total (*n* = 435)	Colombia (*n* = 79)	Ecuador (*n* = 121)	Guatemala (*n* = 80)	Peru (*n* = 155)	*p*-value	V′ cramer
	*n* (%)	*n* (%)	*n* (%)	*n* (%)	*n* (%)		
Age in tertiles							
21–33 years	153 (35.17%)	20 (25.32%)	53 (43.80%)	31 (38.75%)	49 (31.61%)	0.001	0.165
34–41 years	85 (19.54%)	26 (32.91%)	23 (19.01%)	17 (21.25%)	19 (12.26%)		
42–more	197 (45.29%)	33 (41.77%)	45 (37.19%)	32 (40.00%)	87 (56.13%)		
Gender^1^							
Female	295 (67.82%)	58 (73.42%)	92 (76.03%)	48 (60.00%)	97 (62.58%)	0.028	0.114
Male	140 (32.28%)	21 (26.58%)	29 (23.97%)	32 (40.00%)	58 (37.42%)		
Civil status^2^							
Married/cohabitant	249 (57.24%)	39 (49.37%)	87 (71.90%)	50 (62.50%)	73 (47.10%)	<0.001	0.212
Single	161 (37.01%)	38 (48.10%)	21 (17.36%)	30 (37.50%)	72 (46.45%)		
Other	25 (5.75%)	2 (2.53%)	13 (10.74%)	0 (0.00%)	10 (6.45%)		
Number of children^1^							
No children	191 (43.91%)	50 (63.29%)	29 (23.97%)	38 (47.50%)	74 (47.74%)	<0.001	0.130
One child	85 (19.54%)	8 (10.13%)	22 (18.18%)	22 (27.50%)	33 (21.29%)		
Two or more children	159 (36.55%)	21 (26.58%)	70 (57.85%)	20 (25.00%)	48 (30.97%)		
Time working (in years)^3^							
Me (Sd)	7.29 (6.92)	5.36 (4.59)	7.25 (7.08)	5.61 (5.04)	9.18 (8.09)	<0.001	0.117
Mental exhaustion^1^							
No	42 (9.66%)	7 (8.86%)	20 (16.53%)	3 (3.75%)	12 (7.74%)	0.015	0.123
Yes	393 (90, 34%)	72 (91.14%)	101 (83.47%)	77 (96.25%)	143 (92.26%)		
Fear of COVID-19^3^							
Me (Sd)	1.65 (1.04)	1.75 (0.78)	1.52 (0.43)	1.63 (0.76)	1.36 (0.58)	0.237	0.015
Risk perception to COVID-19^3^							
Me (Sd)	3.15 (0.94)	3.41 (1.18)	2.73 (1.57)	3.55 (1.16)	3.14 (1.29)	<0.001	0.152
Preventive behaviours: additional protection at work^3^							
Me (Sd)	7.43 (2.16)	7.99 (1.68)	6.71 (2.48)	7.43 (2.52)	7.72 (1.73)	<0.001	0.156
Preventive behaviours: hand washing^3^							
Me (Sd)	5.59 (1.15)	5.6 (1.12)	5.62 (1.12)	5.60 (1.24)	5.55 (1.16)	0.800	0.032
Preventive behaviours: total^3^							
Me (Sd)	13.02 (2.44)	13.58 (1.92)	12.33 (2.66)	13.03 (2.97)	13.27 (2.05)	<0.001	0.146

Me, mean; SD, standard deviation.

^1^The Chi-square test was used.

^2^The Fisher Exact test was used.

^3^The ANOVA test was used and Omega-squared.

The main variables show that the mean score of fear of COVID-19 according to the total sample was 1.65 (SD = 10.04), risk perception was 3.15 (SD = 0.94). The mean score for additional protective behaviours at work reported was 7.43 (SD = 2.16), hand washing reported was 5.59 (SD = 1.15); meanwhile, the total score for all preventive behaviours was 13.02 (SD = 2.44).Furthermore, it was found that there were significant differences, albeit with low effect sizes, between countries in relation to the scores obtained by health personnel, particularly in terms of age (V′ Cramer = 0.165), risk perception (Omega-Squared = 0.152), and preventive measures (Omega-Squared = 0.146). The risk perception in Ecuador was higher than in Guatemala (*p* < 0.001), while Guatemala reported higher scores than Ecuador (*p* < 0.001). Peruvian health professionals showed higher scores than Ecuadorian professionals in terms of preventive behavior (*p* < 0.001) (see Appendix E).

Table 2 shows the characterization of the main variables of the study, which indicates that more than three quarters of the health professionals showed signs of fear of COVID-19 infection (93.10% were concerned about becoming infected, 95.63% were concerned about returning home and infecting their family, and 88.51% were concerned about the possibility of dying from the disease). Likewise, more than three quarters reported indications of risk perception to COVID-19 disease (e.g., 87.82% had direct contact with suspected or confirmed COVID-19 patients in aerosol generation procedures and 81.15% had direct contact with the environment of confirmed COVID-19 patients). Regarding preventive behavior, more than three fifths showed indications of always using additional protection at work (e.g., 73.56% always used face shield or goggles and 72.97% used gloves for care at work), while more than four fifths of the health personnel reported that they always perform hand washing (e.g., 91.95% performed hygiene after exposure to body fluids of any type of patient).

**Table 2:** Prevalence of fear of COVID-19, risk perception and preventive behavior in health professionals (*n* = 435).

Variables	*n* (%)
Fear of COVID-19	
Are you afraid/concerned that you might become infected?	
No	30 (6.90%)
Yes	405 (93.10%)
Are you afraid/concerned about returning home and infecting your family?	
No	19 (4.47%)
Yes	416 (95.63%)
Are you afraid/concerned that you might die from COVID-19?	
No	50 (11.49%)
Yes	385 (88.51%)
Risk perception	
Have you provided direct care to a confirmed patient with COVID-19?	
No	123 (28.30%)
Yes	312 (71.70%)
Did you have face-to-face contact (within 1 meter) with a confirmed COVID-19 patient in a health care facility?	
No	112 (25.80%)
Yes	323 (74.20%)
Were you present when any aerosol generation procedure was performed on suspected or confirmed cases of COVID-19?	
No	53 (12.28%)
Yes	382 (87.82%)
Did you have direct contact with the environment where the confirmed COVID-19 patient was cared for? For example, bed, bedding, medical equipment, bathroom?	
No	82 (18.95%)
Yes	353 (81.15%)
Preventive practices	
D1: Additional protection at work	
Do you use disposable gloves in the workplace?	
Rarely	34 (7.82%)
Occasionally	54 (12.41%)
Most of the time	30 (6.90%)
Always	317 (72.97%)
Do you use face shield or goggles in the workplace?	
Rarely	21 (4.83%)
Occasionally	32 (7.36%)
Most of the time	62 (14.25%)
Always	320 (73.56%)
Do you wear a disposable gown in the workplace?	
Rarely	38 (8.74%)
Occasionally	49 (11.26%)
Most of the time	38 (8.74%)
Always	310 (71.26%)
D2: Hand washing	
During patient care, do you perform hand hygiene before and after touching the patient even though you use gloves?	
Rarely	9 (2.06%)
Occasionally	23 (5.29%)
Most of the time	36 (8.28%)
Always	367 (84.37%)
Do you perform hand hygiene after exposure to body fluids of patients who were unsuspected or confirmed COVID-19 cases?	
Rarely	7 (1.61%)
Occasionally	18 (4.14%)
Most of the time	10 (2.30%)
Always	400 (91.95%)

### Association between fear of COVID-19, risk perception with preventive behavior

Table 3 in the model adjusted only by country (Model 1) reported that fear of COVID-19 and perception were significantly associated with preventive behavior according to dimensions and total. However, for the final model that included significant covariates (Model 2) the independent variables of fear of COVID-19 and risk perception had a slight decrease in the coefficients of association with respect to preventive behavior. It was evidenced that in health personnel the main exposure variable was fear of COVID-19, reporting that the greater the fear of COVID-19, the greater the preventive behavior of infection to COVID-19 (*B* = 1.75, *p* = 0.039 for total behavior; *B* = 1.11, *p* = 0.046 for additional protection at work; *B* = 1.09, *p* = 0.034 for hand washing). The risk perception of COVID-19 infection had a slight direct relationship with preventive behaviours (*B* = 0.31, *p* = 0.041 for total behavior; *B* = 0.20, *p* = 0.026 for hand washing), with the exception of the preventive behavior of using additional protection at work (*p* = 0.459).

**Table 3:** Association between fear of COVID-19 and risk perception with preventive behavior of health personnel (*n* = 435).

Variable	Preventive behavior					
	Additional protection at work		Hand washing		Overall	
	B (95%IC)	*p*-value	B (95%IC)	*p*-value	B (95%IC)	*p*-value
Model 1 Age in tertiles						
21–33 years	Ref		Ref		Ref	
34–41 years	1.83 (0.45–3.21)	**0.024**	0.19 (−0.28–0.67)	**0.024**	2.03 (0.79–3.26)	**0.014**
42 to more	1.74 (−0.05–3.53)	0.054	0.31 (0.01–0.63)	0.048	2.06 (0.56–3.55)	**0.022**
Gender						
Female	Ref		Ref		Ref	
Male	−0.09 (−0.93–0.75)	0.749	−0.15 (−0.50–0.19)	0.238	−0.25 (−1.37–0.87)	0.526
Civil Status						
Married/cohabitant	Ref		Ref		Ref	
Single	0.17 (−0.41–0.75)	0.42	−0.05 (−0.54–0.44)	0.75	0.12 (−0.42–0.66)	0.539
Other	0.03 (−1.76–1.82)	0.96	0.21 (−0.16–0.58)	0.175	0.24 (−1.87–2.35)	0.745
Number of children						
No children	Ref		Ref		Ref	
One child	1.20 (−0.81–3.21)	0.153	0.25 (−0.62–1.12)	0.428	1.45 (−0.24–3.14)	0.071
Two or more children	2.21 (0.11–4.31)	**0.044**	0.02 (−0.73–0.77)	0.949	2.23 (0.09–4.37)	**0.045**
**Time working (in years)**	0.01 (−0.04–0.45)	0.895	0.01 (0.01–0.17)	**0.033**	0.01 (−0.04–0.06)	0.521
Mental Exhaustion						
No	Ref		Ref		Ref	
Yes	1.74 (0.49–2.99)	**0.021**	−0.27 (−0.58–0.03)	0.065	1.15 (0.16–2.14)	0.034
**Fear of COVID-19**	1.17 (0.68–2.10)	0.042	1.03 (0.80–1.96)	0.037	1.92 (1.53–2.54)	0.023
**Risk perception**	0.37 (0.10–0.63)	0.022	0.23 (0.11–0.35)	0.010	0.60 (0.36–0.83)	0.004
Model 2 Age in tertiles						
21–33 years	Ref		Ref		Ref	
34–41 years	1.48 (0.2–2.76)	0.035	−0.11 (−0.41–0.19)	0.297	1.34 (0.14–2.55)	0.038
42 to more	1.21 (0.14–2.3)	0.037	0.03 (−0.35–0.43)	0.782	1.32 (0.47–2.16)	0.016
Gender						
Female	Ref		Ref		Ref	
Male	–	–	–	–	–	–
Civil Status						
Married/cohabitant	Ref		Ref		Ref	
Single	–	–	–	–	–	–
Other	–	–	–	–	–	–
Number of children						
No children	Ref		Ref			
One child	0.72 (−0.88–2.33)	0.248	–	–	0.65 (−0.2–1.5)	0.093
Two or more children	1.68 (0.63–2.73)	0.015	–	–	1.45 (1.02–1.88)	0.002
**Time working (in years)**			**0.01 (−0.01–0.02)**	**0.079**	–	–
Mental Exhaustion						
No	Ref		Ref		0.42 (−0.62–1.47)	0.288
Yes	1.1 (0.59–1.61)	0.006	–	–	0.42 (−0.62–1.47)	0.288
**Fear of COVID-19**	1.11 (0.85–1.78)	0.046	1.09 (0.68–1.13)	0.034	1.75 (1.35–2.53)	0.039
**Risk perception**	0.15 (−0.35–0.68)	0.459	0.20 (0.05–0.78)	0.026	0.31 (0.03–0.0.62)	0.041

B, unstandardized coefficient; CI, confidence intervals.

^a^Model 1 was the crude model only taking into account the adjustment by country.

^b^Model 2 was adjusted by country and covariates that were significant in the crude model (*p* < 0.05).

The *p*-values in bold are reported to be significant in both model 1 and 2.

## Discussion

This study aimed to examine the association between fear and risk perception of COVID-19 and preventive behavior in health professionals from Latin American countries in order to provide basic data to respond to the mental health problems faced by health personnel in middle-income countries. In the presence of emerging events or conditions, such as the case of the pandemic, health professionals have been required to use their emotional and cognitive resources to ensure adaptive mechanisms in their clinical practice and daily life.

Among the 435 professionals included in the study, 90.34% exhibited mental exhaustion as a result of COVID-19, which is significantly higher than the rates reported in previous studies (5, 28, 29, 36). This difference can be attributed to the fact that the sample was obtained from various Latin American countries, which have been among the hardest hit by the pandemic due to limited resources to address it (37). This situation has led to higher rates of psychological problems among healthcare professionals in the region compared to other parts of the world, such as Europe and Asia (38).

In this study, high frequencies of mental exhaustion were found in each of the countries, a situation that differs from several studies conducted worldwide (9, 39). In addition, it was found that 83.47% of the Ecuadorian health professionals included had mental exhaustion. This also differs from the reports in the available evidence (40, 41). The same difference was found in Peruvian health professionals, where 92.26% had mental exhaustion, which is far from what was previously reported (42). It should be noted that these marked differences between the findings of this study and those reported in the evidence may be mostly due to the type of sampling applied, which does not guarantee the representativeness of the population of health professionals in Colombia, Ecuador, Guatemala, and Peru.

The available literature reports that the main factors associated with mental exhaustion are the inadequate organization and structure of the work, as well as the ability to cope with and manage stressors in COVID-19 care centers (43). Likewise, other studies found that the work overload to which health professionals were subjected during the first waves of the pandemic was a predisposing factor to mental fatigue (44).

Finally, a 2020 study found that the lack of personal protective equipment was associated with mental fatigue, fear of COVID-19 and anxiety symptoms in frontline personnel (45). We should keep in mind that, according to the World Health Organization, workers who do not receive enough support and who have limited control over how they can cope with work demands are more likely to have work-related stress, which affects their mental health and performance (46). These associated factors reported in the cited studies may explain the increased risk perception to COVID-19 experienced by frontline care professionals. This relates to what was found in this study since more than 75% of the included professionals reported indications of risk perception to COVID-19 disease; furthermore, the association between fear of COVID-19 and risk perception has been previously reported (15). However, unlike our study, the studies cited were conducted in a single country, so it is recommended to conduct multicenter studies to assess whether these risk factors for mental exhaustion are present in more Latin American countries for a better understanding of the problem.

An overall mean fear of COVID-19 scores of 1.80 was found, with Colombia being the country that had the highest average with 1.89, while for Peru the average was 1.84. Besides, the average overall risk perception score was 3.15, with Guatemala being the country with the highest average with 3.55, while Peru had 3.14 These results show the high levels of fear and risk perception present among health personnel, which has been previously reported (4, 47), where high levels of fear of getting COVID-19 or infecting family members, risk perception and death were found. We did not find studies evaluating these rates in Latin American countries; however, a study that included dentists from all over the world evaluated the fear of COVID-19 experienced by these professionals and found that more than 78% reported that they do feel it (48), which reinforces what was found in this study.

It was found that the average global score for preventive behaviours was 13.02 and that Colombia had the highest average. Also, the use of additional protection at work was the preventive behavior with the highest average score, and Colombia and Peru were the countries with the highest scores. Due to several research in the area, it has been established that the incorporation of preventive measures such as hand washing, the use of masks and face shields are the main and most effective measures for preventing COVID 19 infection. In this regard, external factors such as the dissemination and training in the use of clinical practice guidelines, the dissemination of information in institutional and mass media and the availability of supplies in the workplace have an impact on the incorporation of protective measures during care in clinical scenarios (49).

This study found that 93.1% reported feeling fear of becoming infected, while 95.63% felt fear of infecting their family. This coincides with what has been reported in other studies, where they found that the main fear of health professionals was to return home and infect their family, followed by the fear of becoming infected (39, 50, 51). This reaffirms the fact that health personnel are exposed to multiple stressors and concern factors, where the most affected are the personnel who work in the first line of care against COVID-19, making transit to other scenarios of the daily life of this population (18).

Furthermore, we found an association between fear of COVID-19 and the risk perception with preventive behaviours, where the greater the fear or perceived risk perception, the greater the attitude of taking preventive actions, as reported in other studies (15, 47, 49, 52).

This study has limitations: (1) The sampling applied was non-probabilistic, which does not guarantee the representativeness of the study population of the countries included and, therefore, it is not possible to extrapolate the results (2) Since an online survey was applied to report mental exhaustion, the result is subject to the subjectivity of the person completing the survey for no test or diagnostic procedure was applied. (3) Another limitation was the development of the questionnaires for this study, which were based on the policies proposed by the World Health Organization of COVID-19 for use in the work of health professionals. Although questionnaires for many of our study variables already exist, such as the Fear of COVID-19 Scale (35) and Preventive COVID-19 Infection Behaviours Scale (53), it was taken into account that, during the research development period, there were no brief questionnaires specifically designed for our study population in the work and in the context of the pandemic. Nevertheless, despite the limitations, this study is relevant because it is one of the first to report the rates of fear, risk perception, and preventive behaviour in health professionals in Latin American countries.

In conclusion, we found that fear and risk perception are associated with increased practice of hand washing and use of additional protection at work. Nevertheless, further studies on the subject are needed because working conditions during the pandemic greatly influence the work performance and mental health of frontline staff in the face of COVID-19; therefore, a better understanding of the subject will allow better decisions to be made and avoid medium- and long-term complications for the health care system in Latin America.

## Data availability statement

The raw data supporting the conclusions of this article will be made available by the authors, without undue reservation.

## Ethics statement

The studies involving human participants were reviewed and approved by the information protocol was approved by the ethics committee of the Norbert Wiener University issued in the Register Report No. 085-2020. The participants provided their written informed consent to participate in this study.

## Author contributions

OR-L, CB-A, and IR-L: conceptualization and supervision. OR-L, CB-A, IR-L, EC-A, and MI-Z: methodology. RL, CB-A, IR-L, EC-A, MI-Z, and RP-L: formal analysis. OR-L, CB-A, IR-L, EC-A, RP-L, MI-Z, FC, and LS: investigation, writing—original draft preparation, and writing—review and editing. OR-L and CB-A: project administration. All authors have read and agreed to the published version of the manuscript.

## Funding

This research was financed by the Norbert Wiener University as part of its policy of competitive funds to support research from the Office of the Vice President for Research (N°0622-2023).

## Conflict of interest

The authors declare that the research was conducted in the absence of any commercial or financial relationships that could be construed as a potential conflict of interest.

## Publisher’s note

All claims expressed in this article are solely those of the authors and do not necessarily represent those of their affiliated organizations, or those of the publisher, the editors and the reviewers. Any product that may be evaluated in this article, or claim that may be made by its manufacturer, is not guaranteed or endorsed by the publisher.
