# Supplementary material for: Fear of COVID-19, risk perception and preventive behavior in health workers: a cross-sectional analysis in middle-income Latin American countries
Source: Front Public Health. 2023 Jun 15;11:1171246. doi: 10.3389/fpubh.2023.1171246 (PMC10307953; doi:10.3389/fpubh.2023.1171246)
Supplement: Supplementary file 1 [file Data_Sheet_1.docx]

Supplementary Material

Low infection rate of the SARS-CoV-2 Omicron variant in patients with inflammatory bowel disease: a large-scale retrospective vaccination analysis of three unique cohorts

Jing Feng^1^, Tian Yang^1^, Ruchen Yao^1^, Bo Feng^2,3^, Renshan Hao^3,4^, Yuqi Qiao^1^, Jinlu Tong^1^, Jun Shen^1,3*^

*** Correspondence:** Jun Shen

Email: [shenjun79@sina.cn](mailto:shenjun79@sina.cn)

# Supplementary Tables

Table S1: Comparison of vaccination and characteristics between asymptomatic and healthy individuals.

|  | **IBD (n=420)** | **Healthy (n=896)** | **Asymptomatic (n=889)** | ***P*** |
| --- | --- | --- | --- | --- |
| Age | 34.4 (11.1) | 35.7 (8.82) | 36.2 (8.73) | 0.004 |
| Male | 278 (66.2%) | 489 (54.6%) | 548 (61.6%) | <0.001 |
| Vaccination | 215 (51.2%) | 861 (96.1%) | 651 (73.2%) | <0.001 |
| Dose |  |  |  | <0.001 |
| 1 | 15 (3.57%) | 11 (1.23%) | 29 (3.26%) |  |
| 2 | 90 (21.4%) | 162 (18.1%) | 236 (26.5%) |  |
| 3 | 110 (26.2%) | 688 (76.8%) | 386 (43.4%) |  |

Values represent the mean (standard deviation) or n (%).

Table S2: Incidence of adverse events in vaccinated patients with IBD and healthy individuals

|  | **All (n=1076)** | **IBD(n=215)** | **Healthy(n=861)** | **OR** | ***P*** |
| --- | --- | --- | --- | --- | --- |
| Age | 35.3 (9.39) | 33.5 (11.2) | 35.7 (8.83) | 1.03 [1.01; 1.04] | 0.009 |
| Male | 627 (58.3%) | 155 (72.1%) | 472 (54.8%) | 0.47 [0.34; 0.65] |  |
| Dose |  |  |  |  | <0.001 |
| 1 | 26 (2.42%) | 15 (6.98%) | 11 (1.28%) | Ref. |  |
| 2 | 252 (23.4%) | 90 (41.9%) | 162 (18.8%) | 2.44 [1.07; 5.71] |  |
| 3 | 798 (74.2%) | 110 (51.2%) | 688 (79.9%) | 8.46 [3.78; 19.5] |  |
| Adverse events | 146 (13.6%) | 31 (14.4%) | 115 (13.4%) | 0.91 [0.60; 1.42] | 0.768 |

Values represent the mean (standard deviation) or n (%).
